# Insights into the Electrochemical Reduction of 5‐Hydroxymethylfurfural at High Current Densities

**DOI:** 10.1002/cssc.202102504

**Published:** 2022-03-03

**Authors:** Giancosimo Sanghez de Luna, Adriano Sacco, Simelys Hernandez, Francesca Ospitali, Stefania Albonetti, Giuseppe Fornasari, Patricia Benito

**Affiliations:** ^1^ Dip. di Chimica Industriale “Toso Montanari” University of Bologna Viale Risorgimento 4 40136 Bologna (BO) Italy; ^2^ Center for Sustainable Future Technologies @POLITO Istituto Italiano di Tecnologia Via Livorno 60 10144 Turin Italy; ^3^ Department of Applied Science and Technology (DISAT) Politecnico di Torino C.so Duca degli Abruzzi 24 10129 Turin Italy

**Keywords:** 2,5-bishydroxymethylfuran, 5-hydroxymethylfurfural, AgCu, electrocatalysis, galvanostatic electrochemical reduction

## Abstract

The electrocatalytic reduction of 5‐hydroxymethylfurfural (HMF) is highly selective to 2,5‐bishydroxymethylfuran (BHMF) at pH=9.2, diluted HMF solutions, and low current densities. In this work, the electrochemical reduction of 0.05 m HMF solutions was investigated in the 5–50 mA cm^−2^ current density range over an AgCu foam electrocatalyst. The selectivity towards the formation of BHMF or the dimerization depended on the current density, likely due to differences in the electrode potential, and on the reaction time. Operating at current densities of 40–50 mA cm^−2^ allowed to find a trade‐off between HMF and H_2_O activation, achieving 85 % BHMF selectivity and fostering the productivity (0.567 mmol cm^−2^ h^−1^), though co‐producing H_2_. The electrochemical characterization by Tafel slopes and electrochemical impedance spectroscopy indicated that the HMF reduction was kinetically favored in comparison to the hydrogen evolution reaction and that the process was limited by charge transfer.

## Graphical abstract

**BHMF electrosynthesis**: The electrocatalytic reduction of 0.05 m 5‐hydroxymethylfurfural (HMF) solutions to 2,5‐bishydroxymethylfuran (BHMF) is feasible at high current densities over nanostructured AgCu foam electrocatalysts. High BHMF selectivity and productivity values are achieved, suppressing the formation of hydrofuroin by‐product, though co‐producing H_2_. The process is fostered in the potential window where HMF reduction is favored and H_2_ production from the hydrogen evolution reaction is not inhibited.

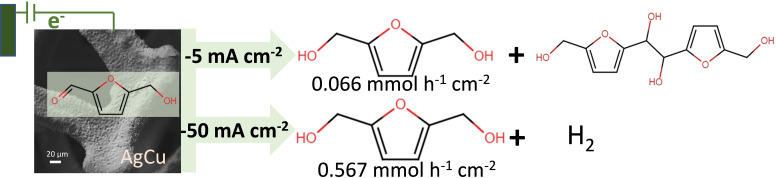

## Introduction

5‐Hydroxymethylfurfural (HMF) is a biomass‐derived platform molecule largely investigated for the production of chemicals and fuels.[^1^, ^2^] 2,5‐Bishydroxymethylfuran (BHMF, a polymer precursor) and 2,5‐dimethylfuran (DMF, a fuel additive) are obtained by thermocatalytic hydrogenation of HMF at high H_2_ pressure in autoclave reactors.^[3]^ To increase the sustainability of the process, the replacement of H_2_ by bio‐alcohols (hydrogen transfer) has been proposed.[^4^, ^5^] Another alternative that is gaining increasing interest in the conversion of biomass‐derived compounds, among them HMF, is the electrocatalytic hydrogenation (ECH).[^6^, ^7^, ^8^] This electrochemical approach operates at room temperature and atmospheric pressure, and the electrons come from renewable electricity, while the H^+^ is supplied by water. Though highly promising, the electrochemical conversion of HMF is still in its infancy and more knowledge is required to enhance the productivity, the key parameter for its deployment.

**Scheme 1:**
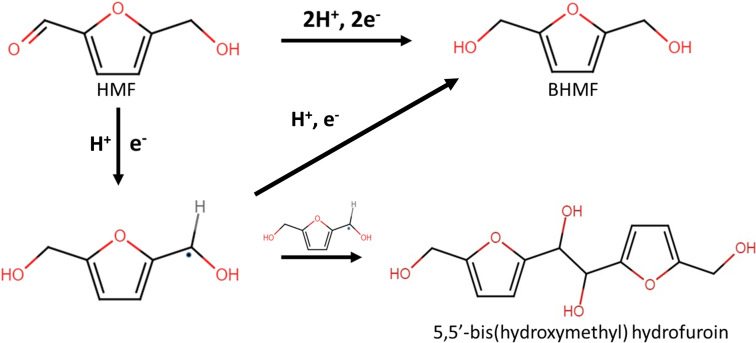
Possible reaction pathways during the electroreduction of HMF.

A wide range of HMF reduction products can be obtained depending on the type of electrocatalyst, applied potential, pH, and composition of the electrolyte. Broadly speaking, in acidic media, DMF,[^9^, ^10^, ^11^] tetrahydrofuran,^[10]^ or ring‐opening products such as hexanedione^[12]^ can be produced, while turning to a basic media and using poor hydrogen evolution reaction (HER) catalysts such as Pb,^[9]^ Cu, and Ag,[^13^, ^14^, ^15^, ^16^] the selectivity shifts to the bisalcohol BHMF (Scheme 1). The concentration of the substrate is another important parameter in the electrochemical reaction. In acidic media, the coulombic efficiency of the HMF hydrogenation is reported to increase from 25 to 55 % at starting concentrations of 0.05 and 0.50 m, respectively.^[9]^ On the contrary, at pH=9.2 the selectivity in BHMF drops when moving from 0.02 to 0.50 m HMF electrolytes due to hydrodimerization (Scheme 1) and possibly polymerization reactions.[^13^, ^14^, ^15^, ^16^] The applied potential also contributes to drive the reduction to the desired product. For instance, the contribution of the hydrodimerization in the electrochemical conversion of diluted HMF solutions (0.02 m) is decreased at more negative potentials than −1.3 V vs. Ag/AgCl over Ag/C electrocatalysts.^[15]^ On carbon electrodes (without any metal species), the same trend is observed, but the production of the dimer is fostered, even with very diluted HMF solutions (e. g., 0.01 m).^[17]^

To explain the differences in the selectivity of the electrochemical HMF reduction, the reaction mechanism should be considered. Two general reaction pathways have been proposed for the electroreduction of aromatic and furanic aldehyde molecules: the proton‐electron (H^+^/e^−^) transfer and the reaction with H_ads_.[^18^, ^19^, ^20^, ^21^] In the former, the H^+^ is taken directly from the water and the electron transfer could occur either through inner or outer shell spheres;^[20]^ the individual H^+^/e^−^ steps could take place simultaneously or consecutively. Conversely, in the H_ads_ route, an initial Volmer step is required to activate the H^+^ or H_2_O on the electrocatalyst surface. The reaction of the aldehyde group with one H^+^/e^−^ pair generates a radical intermediate; it can either dimerize with a second radical or be further converted with another H^+^/e^−^ to yield the alcohol as product. Alternatively, the first coupled H^+^/e^−^ transfer could be followed by an inner sphere reaction with H_ads_.[^22^, ^23^] These electrochemical reaction pathways depend on the type of electrocatalyst and the pH of the electrolyte. The latter not only promotes or inhibits the Volmer step,^[23]^ but also the H^+^/e^−^ transfer sequence,[^24^, ^25^] the polarizability of the aldehyde, and in turn its interaction with the electrocatalyst.[^19^, ^26^]

In the HMF electroreduction in neutral media, H_2_O is likely the hydrogen source for the conversion of HMF to BHMF.^[27]^ Conversely, both the hydrogenolysis of HMF to DMF in acidic media^[10]^ and the reduction of the aldehyde to the alcohol (BHMF) in basic media have been hypothesized to occur through the involvement of H_ads_.[^13^, ^14^, ^15^, ^17^] However, there are some discrepancies in the potential window for the H_ads_‐mediated pathway at pH=9.2 on Ag electrodes, which could be either within^[13]^ or outside^[15]^ the region of the HER. Furthermore, the formation of hydrofuroin by electrohydrodimerization in basic media means that the H^+^/e^−^ pathway also occurs, though it is not clearly stated. Hence, both H^+^/e^−^ and the H_ads_ routes are in competition on Ag and Cu; the electrocatalyst coverage by HMF or intermediate molecules and H_ads_ likely determines the pathway and therefore the selectivity.[^14^, ^15^]

To the best of our knowledge, the feasibility of the HMF electroreduction to operate at high current densities is limited to one work [280 mA cm^−2^ with Ag nanoparticles (NPs)/carbon cloth (CC) and 0.10 m HMF solution],^[28]^ notwithstanding the importance of the current density in the deployment of the process. For instance, an optimal current density usually exists for ECH of organic compounds.^[29]^ This parameter, like the substrate concentration, may alter the mechanism and therefore the selectivity, altering the product distribution due to concentration overpotential as observed for the CO_2_ electroreduction.^[30]^ Additionally, the deactivation due to the formation of polymeric compounds could not be discarded under harsh reaction conditions, as previously observed for furfural.[^23^, ^31^]

The aim of this work is to gain insight into the electrochemical conversion of 0.05 m solutions of HMF into BHMF at increasing applied current densities over a highly active AgCu 3D foam catalyst previously developed by us.^[32]^ The use of this nanostructured and 3D catalyst largely increases the productivity; however, a larger electroactive surface area does not suppress the formation of the hydrofuroin by‐product when the concentration of HMF in the electrolyte is increased. To achieve the goal of the work, the current density applied is varied in the range between 5 and 50 mA cm^−2^ (note that the area used to calculate the surface area corresponds to both faces of the foam piece). The values of BHMF selectivity, faradaic efficiency (FE), and productivity are correlated to the potential applied, the kinetics of the HMF reduction and HER (estimated by Tafel plots), as well as to charge and mass transfer properties [obtained from electrochemical impedance spectroscopy (EIS) measurements]. Moreover, to modify the activity in the HER, the pH of the electrolyte is changed, and Ni catalysts are tested. The results obtained suggest some reaction conditions to maximize either the radical coupling or the electrochemical reduction.

## Results and Discussion

**Figure 1:**
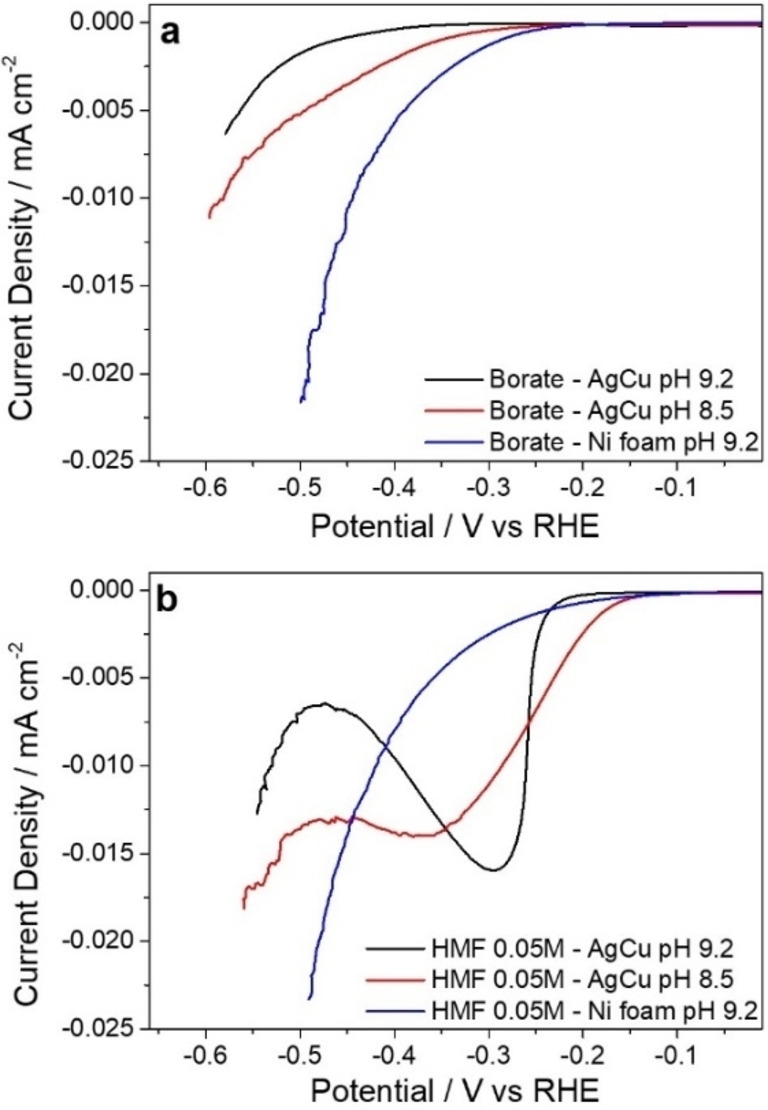
LSVs in (a) borate and (b) 0.05 m HMF+borate over an AgCu foam at pH 9.2 and 8.5 and over a Ni foam at pH 9.2. Range: 0 to −0.61 V vs RHE. Scan rate: 1 mV s^−1^ for borate solutions and 5 mV s^−1^ for HMF‐containing solutions.

The electrocatalytic tests are performed over an AgCu foam previously investigated by us.[^16^, ^32^] Briefly, the electrocatalyst is made by arrays of bimetallic AgCu nanoparticles that evenly coat the foam surface, few dendrites develop in the pore and strut edges. Over this catalyst, the characteristic HMF reduction peak is observed in linear sweep voltammetry (LSV) at pH=9.2 for the 0.02–0.10 m HMF concentration range. The onset of HMF reduction is around 60 mV lower than the HER onset (see Figure 1 and Refs. [16,32]).

### Electrocatalytic reduction tests of HMF at fixed current density and potential

**Figure 2:**
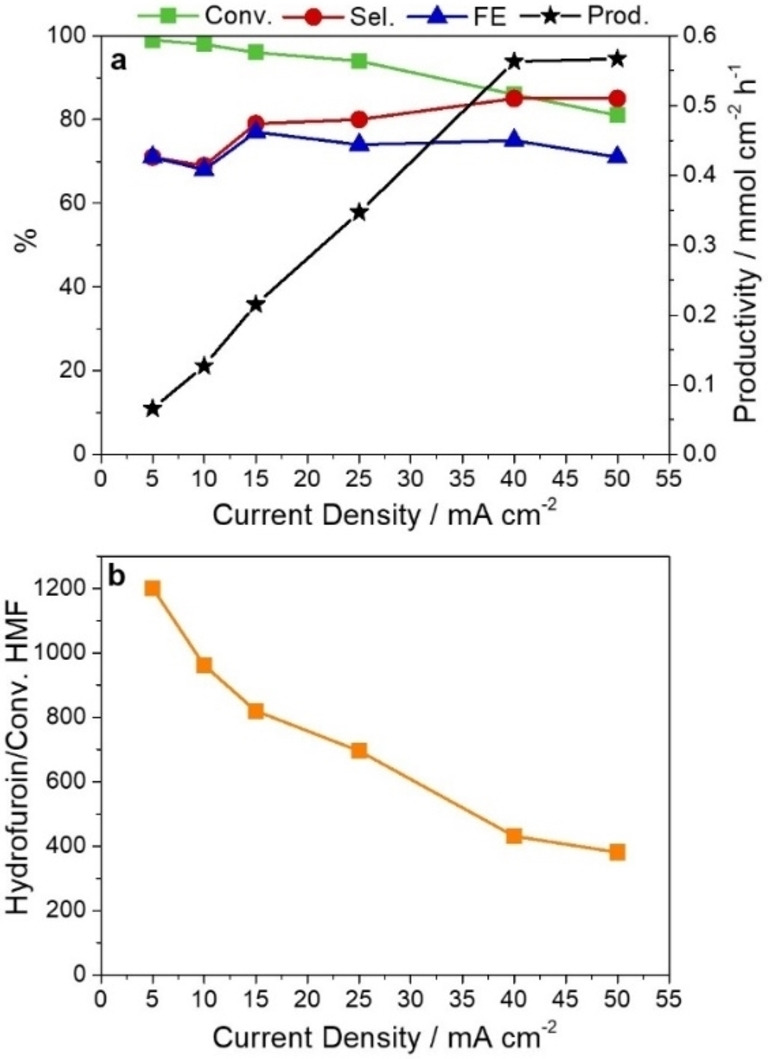
Effect of the current density on (a) HMF conversion, BHMF selectivity, productivity, and FE, and (b) hydrofuroin formation. Catalyst: AgCu foam; electrolyte: 0.05 m HMF at pH=9.2.

The activity of the catalyst in the electrocatalytic reduction of HMF is investigated in the 5–50 mA cm^−2^ applied current density range (Figure 2). The electrochemical reactions are carried out until the theoretical charge to convert the 1.25 mmol of HMF in the electrolyte to BHMF is accumulated (assuming a 2 e^−^ process and 100 % FE).

Operating at high current densities has a positive impact on the selective formation of BHMF, but a negative effect on the HMF conversion (Figure 2a). Namely, the selectivity in BHMF shifts from 71 to 85 % and the conversion of HMF drops from 99 to 81 % as the current density increases from 5 to 50 mA cm^−2^. These behaviors are likely related to the suppression of the hydrodimerization reaction (see the decrease of the hydrofuroin peak in Figure 2b), and the fostering of the HER by increasing the current density. An excellent 0.567 mmol cm^−2^ h^−1^ BHMF productivity rate is achieved at current densities of 40 and 50 mA cm^−2^. The trade‐off between selectivity and conversion makes the FE values remain rather constant (≈70–75 %) in all the tests. Note that the C balance follows the BHMF selectivity trend (Table S1); it increases from around 71 to 88 %, corresponding to a decrease of hydrofuroin production (from 1201 to 381, expressed as hydrofuroin area/conversion). Hence, the lack of carbon balance can be related to the formation of the dimer, but the presence of oligomers is not discarded.

**Figure 3:**
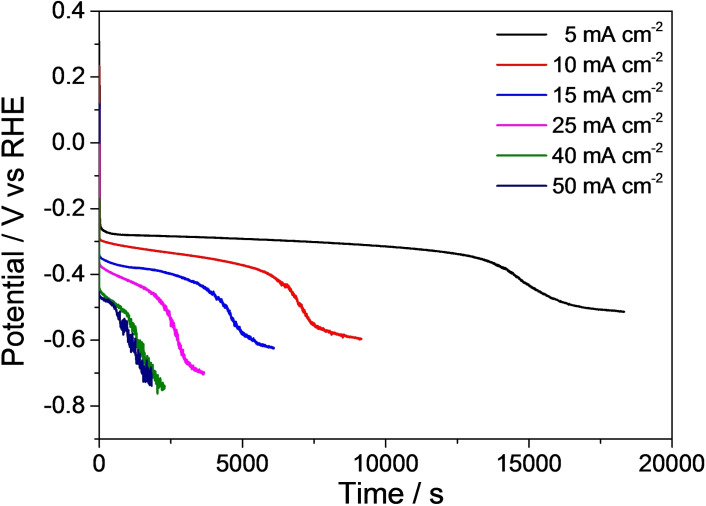
Evolution of the potential with the reaction time during the electrocatalytic reduction of 0.05 m HMF, pH=9.2, over an AgCu foam at different current density values.

Notably, the AgCu foam electrocatalyst enables to reach the set current densities at electrode potentials in the −0.26 to −0.51 V vs. reversible hydrogen electrode (RHE) range, at the beginning of the electroreductions (Figure 3). The concentration polarization drives the potential to the HER region with the lengthening of the reaction; this is confirmed by a test at 5 mA cm^−2^ current density in an electrolyte without HMF (Figure S1a). The higher the applied current density is, the faster the HER becomes significant, since the HMF concentration drops more rapidly. However, by analyzing the HMF conversion and BHMF selectivity values obtained in a test stopped before the concentration polarization region (Figure S1b), it is evidenced that in that last part of the electrochemical test, HMF conversion and, more remarkably, the BHMF selectivity increase. These results indicate that HMF reduction and HER can occur simultaneously.

**Figure 4:**
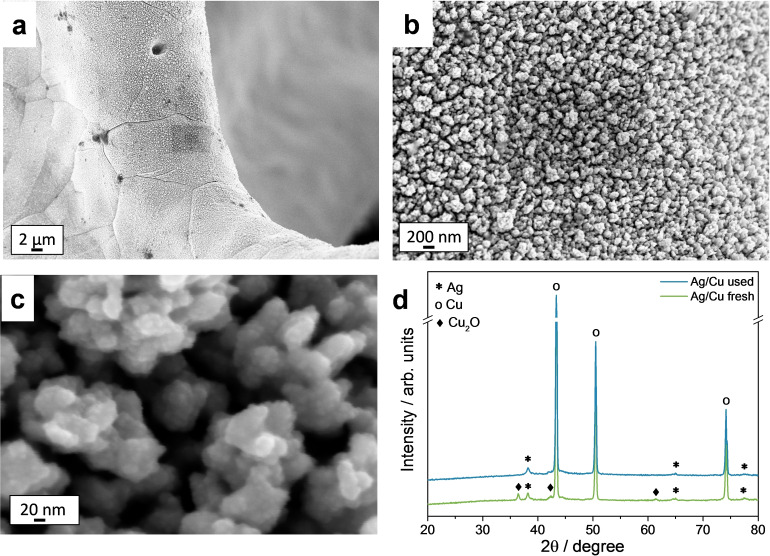
Characterization of the AgCu foam electrocatalyst after 8 h reaction in the 5–50 mA cm^−2^ current density range: (a–c) FE‐SEM images; (d) XRD pattern of the catalyst before and after tests.

The aforementioned tests are performed over the same AgCu foam electrocatalyst, also carrying out in between tests LSVs in borate without and with HMF. Field‐emission scanning electron microscopy (FE‐SEM) images indicate that, after around 8 h of electrochemical tests, and reaching in some parts of the experiments potentials as high as −0.74 V vs. RHE, the coating remains well‐adhered to the foam surface (Figure 4a, b). However, an inspection of the surface at high magnification (Figure 4c) reveals that in the arrays the particles are more rounded and interconnected than before tests, suggesting their sintering. We have previously observed that the sintering is accompanied by a Cu‐enrichment.^[32]^ X‐ray diffraction (XRD) patterns of the spent catalyst confirm the presence of Ag^0^. The Cu_2_O phase, observed in the fresh catalyst, disappears (Figure 4d).

Taking into account the changes in the electrocatalyst during reaction, the stability and reproducibility of the results herein presented are investigated. A series of experiments are carried out modifying the sequence of the runs. When performing three consecutive tests at 5 and 15 mA cm^−2^ current density values over the same catalyst for each set value (Figure S2a, b), the potential required to keep the same current density slightly increases. Moreover, the deposition of carbonaceous species is observed in SEM images (Figure S2c), which could be related to the formation of polymeric compounds. Remarkably, while in the set of tests at current density of 5 mA cm^−2^ the potential steadily increases from the first to the third cycle, at 15 mA cm^−2^ the differences are only recorded between the first and second cycle. Nonetheless, the small changes in the potential do not significantly alter the catalytic activity.

**Figure 5:**
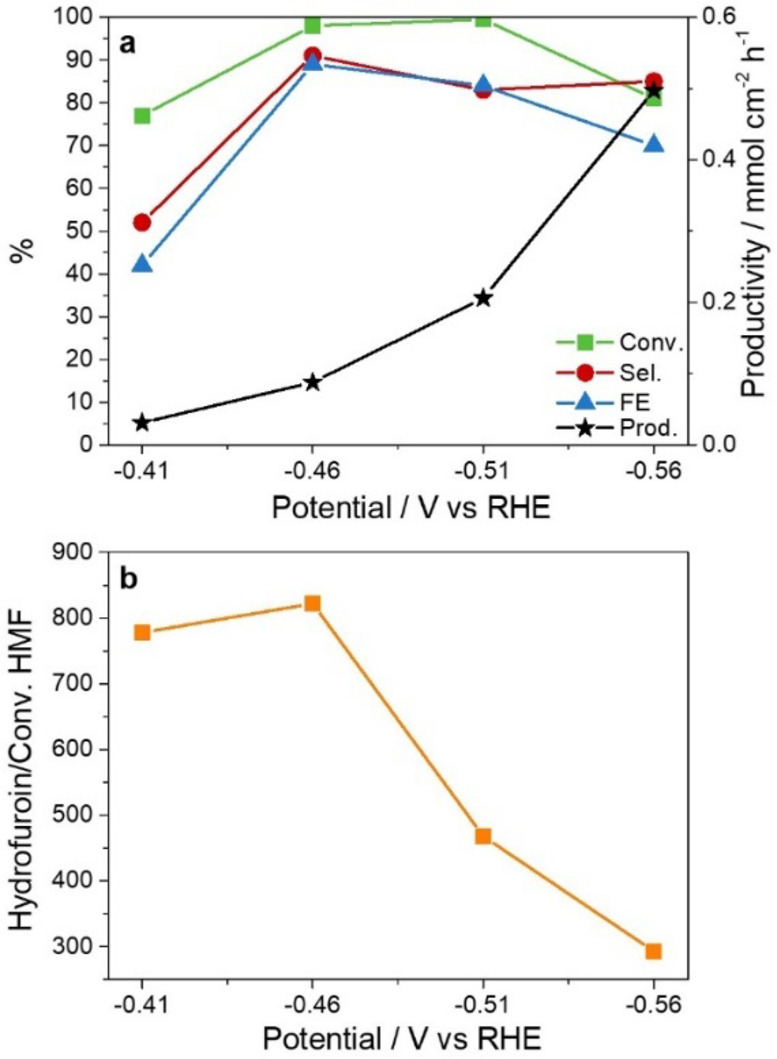
Effect of the potential applied on (a) HMF conversion, BHMF selectivity, productivity, and FE, and (b) formation of hydrofuroin. 0.05 m HMF electrolyte, pH=9.2, AgCu electrocatalyst.

The results obtained with the 0.05 m HMF solution are in line with those previously reported for a more diluted 0.02 m HMF electrolyte.^[15]^ They underline the role of the electrode potential on the HMF electrocatalytic reduction. At low applied current density values, since the AgCu catalyst operates at a low overpotential, the electrodimerization occurs, while the selectivity in BHMF increases at electrode potentials wherein the HER is also taking place. The potential‐dependent behavior is further confirmed by electroreduction of the 0.05 m HMF electrolyte at applied constant potentials in the −0.41 to −0.56 V vs. RHE range (Figure 5). The selectivity in BHMF increases at potentials equal or more cathodic than −0.46 V vs. RHE (Figure 5a). Moreover, the values of area of the hydrofuroin peak normalized by the HMF conversion are modified with the applied potential, around 780, 820, 480, and 300 at −0.41, −0.46, −0.51, and −0.56 V vs. RHE, respectively (Figure 5b). It should be noted that the reaction at −0.41 V was not completed due to the long time required to collect the charge. Furthermore, as the cathode potential increases above −0.51 V vs. RHE, the lower dimer production does not correspond to a greater selectivity in BHMF, suggesting that other side reactions are taking place (e. g., hydrogenolysis), which are under current investigation.

**Figure 6:**
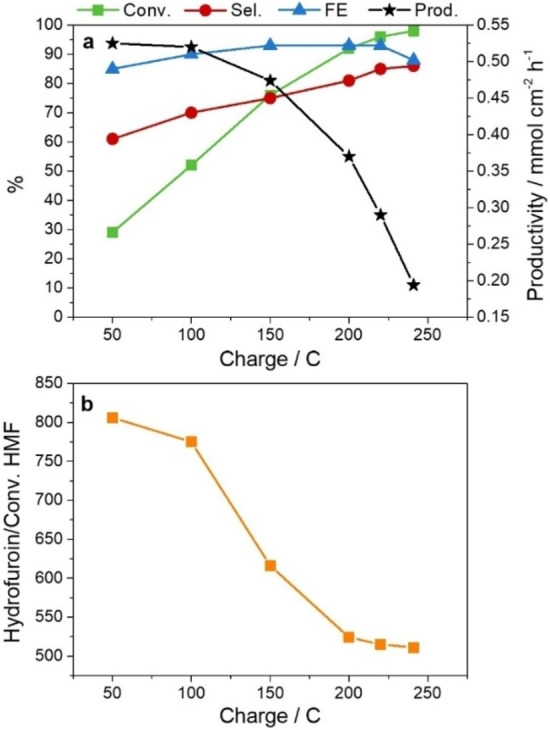
Evolution of (a) HMF conversion, BHMF selectivity and productivity, and FE, and (b) hydrofuroin formation with the charge accumulated during a test at −0.51 V vs. RHE with a 0.05 m HMF electrolyte and an AgCu electrocatalyst.

In this work we observe that the BHMF selectivity is not only potential‐ but also charge (or time)‐dependent. The BHMF selectivity increases with the reaction time during tests at constant current and potential. For instance, in a test applying a potential of −0.51 V vs. RHE (Figure 6), the BHMF selectivity increases from 60 to above 80 % after the accumulation of 50 and 225 C, respectively, and specifically once the hydrofuroin production rate is reduced. These data could explain the higher BHMF selectivity obtained in comparison to the work reported by Chadderdon et al.,^[15]^ since they stopped the reaction at 30 min. Moreover, they again highlight the importance of the HMF concentration on the process selectivity. Actually, two tests with a 0.02 m HMF solution at current densities of 20 and 40 mA cm^−2^ prove that it is possible to reach 90 % selectivity in BHMF (Table S2). Note, however, that a significant amount of charge is used for the H_2_ production, decreasing both conversion and FE, a behavior more remarkable at a current density of 40 mA cm^−2^.

The high selectivity attained even during the reductions at current densities of 40 and 50 mA cm^−2^, for both 0.02 and 0.05 m HMF electrolytes, also indicates that consecutive BHMF electroreduction is not feasible under the reaction conditions investigated, a behavior previously reported in neutral media.^[27]^ The stability of the BHMF in the reaction media is confirmed by an electroreduction performed at an applied potential of −0.51 V vs. RHE with a 0.05 m BHMF solution (Figure S3). Only 6 % of BHMF is converted, and unfortunately the reaction products could not be identified. The current density is similar to the one recorded in a test with only a borate electrolyte, indicating that HER is the main reaction taking place.

The stability of the BHMF molecule may represent an advantage to operate the process in a batch reactor; hence the effect of the electrolyte volume in the performance is investigated at −0.51 V vs. RHE (Figure S4). By doubling the volume of the 0.05 m HMF electrolyte (i. e., 50 mL), keeping the same dimensions of the catalyst, the electrochemical performance (HMF conversion, BHMF selectivity, and FE) is not significantly altered either at 100 C or full charge (241 C).

### Electrochemical characterization and reaction mechanism

The dependence of the selectivity on the electrochemical parameters is likely related to factors that are strongly linked: the electrocatalyst surface coverage, the potential for the activation of HMF and HER, and/or mass transport limitations. For instance, the surface coverage by HMF molecules or reaction intermediates likely grows at high HMF bulk concentration. Moreover, the application of a high cathodic overpotential fosters both the HMF activation and the HER. The HER increases the H_ads_ coverage, which could influence both the HMF reduction pathway involving H_ads_ and the HMF coverage.

Kloth et al. stated that the dimer production for carbon electrodes occurs through an outer‐shell mechanism.^[17]^ An increase in the initial HMF concentration favors the dimer production, which can lead to the depletion of protons near the electrode surface, suppressing the formation of H_ads_, and in turn HER and BHMF production. Chadderdon et al. reported that an imbalance between the mass transfer of HMF to the electrode and its consumption rate may deplete the HMF molecules at the surface of the electrode.^[15]^ Note that the mechanism could be even more complex if it is considered that the coverage degree could modify the orientation of the HMF molecule, and in turn the reactivity, as reported for furfural.^[19]^ Moreover, an increase in surface charge may modify the composition at the electrode/electrolyte interface. Cantu et al. observed that at high electrode charges, the aldehyde population near the surface drops.^[19]^ In contrast, the population of water species increases, indicating that the interfacial environment becomes increasingly hydrophilic at more reductive potentials, causing the organic species to diffuse away from the electrode surface. In this work, to gain insight into some of these aspects, kinetics and mass transfer issues are investigated.

Tafel slope values (≈40–50 and 90–100 mV dec^−1^ in HMF and borate electrolytes, respectively) indicate that the charge transfer occurs faster for HMF reduction than HER. Note, however, that the Tafel slopes are modified during reaction, mainly after the first test at 5 mA cm^−2^ current density; for instance, the HER slope reaches a value close to 140–150 mV dec^−1^ and the HMF slope slightly decreases by around 5–10 mV dec^−1^.

**Figure 7:**
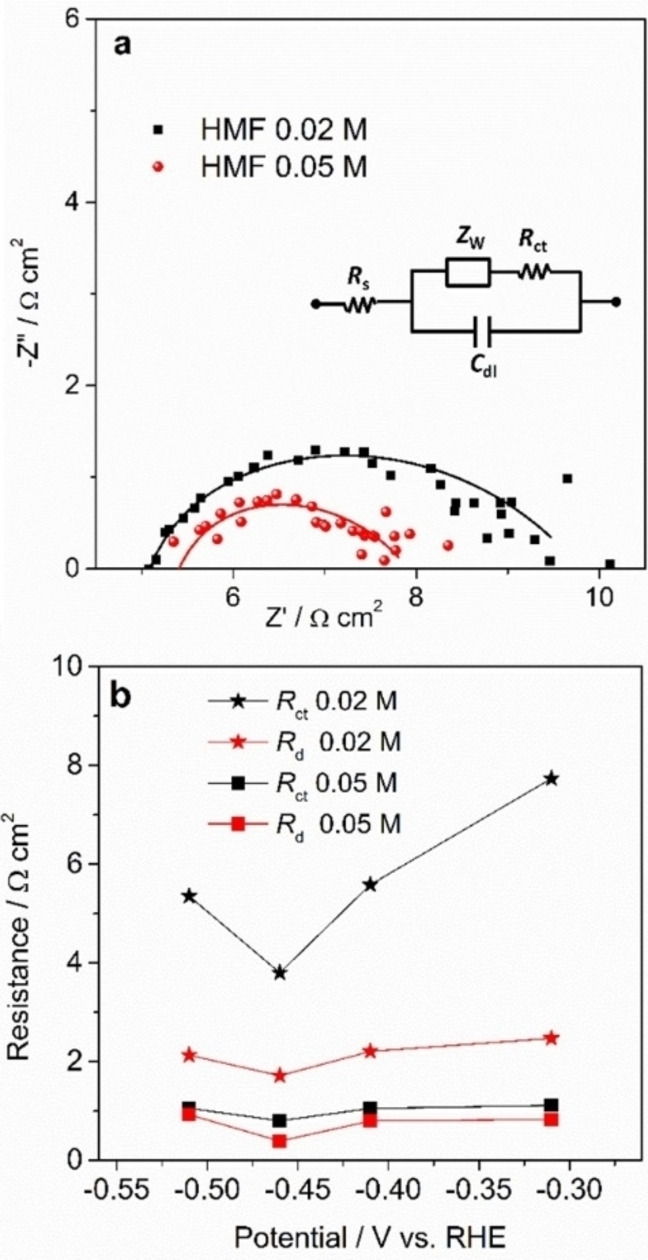
(a) Nyquist plot of AgCu catalyst acquired at −0.51 V vs. RHE in 0.02 and 0.05 m HMF electrolytes (the points refer to experimental data, while the lines refer to the fit obtained by using the equivalent circuit reported in the inset). (b) Charge transfer and diffusion resistances as a function of the applied potential in 0.02 and 0.05 m HMF electrolytes.

To analyze the mass transport properties, EIS measurements have been carried out. Typical Nyquist plots acquired in 0.02 and 0.05 m HMF electrolytes are reported in Figure 7a. All the spectra are characterized by a high‐frequency large arc that is associated to the charge transfer resistance (*R*_ct_) at catalyst/electrolyte interface and a low‐frequency feature related to the mass transport (diffusion) resistance (*R*_d_); the distance from the origin of the axis is linked to the electrolyte resistance (*R*_s_).^[33]^ The extent of the resistance related to charge transfer and mass transport processes has been evaluated by fitting the experimental data through the equivalent circuit shown in the inset of Figure 7a, where *C*_dl_ is the double layer capacitance and *Z*_w_ is the Warburg impedance, related to *R*_d_.^[34]^ This operation has been repeated for the potentials in the range −0.41 to −0.51 V vs. RHE, and the results are shown in Figure 7b. This analysis reveals that *R*_ct_ is larger than *R*_d_ in all the investigated range and for both the concentrations, implying that no mass transfer issue occurs, and the charge transfer is the limiting process.

Moreover, *R*_ct_ depends on the applied potential; it decreases as the electron flow increases, the effect being more remarkable for the 0.02 HMF electrolyte. Note that the minimum of *R*_ct_ is reached at −0.46 V vs. RHE, corresponding to the maximum in the performance as reported in Figure 5. At −0.51 V vs. RHE the *R*_ct_ increases in agreement with a higher contribution of the HER.

In summary, the differences in the activity depending on the potential applied and reaction time, which are also HMF‐concentration dependent, could not be related to mass transfer issues but to charge transfer limitations. At pH=9.2, the electroreduction of HMF is favored by both kinetics and thermodynamics, that is, the onset of the HMF reduction occurs at a lower overpotential that the onset of the HER, and the Tafel slope is smaller in presence of HMF. This could explain the possibility to reach around 100 % FE at −0.51 V vs. RHE, an electrolysis potential more negative than the onset of the HER.[^14^, ^16^]

To account for the decrease in the BHMF selectivity at increasing HMF concentration the trade‐off potential–concentration should be considered. At a low current density and 0.05 m HMF concentration, the electrode potential is around −0.25/−0.30 V vs. RHE. This potential is high enough to activate the aldehyde group through the H^+^/e^−^ pathway, but it is lower than the one required to activate the HER (e. g., −0.50/−0.55 V vs. RHE to reach a current density of 5 mA cm^−2^ in the borate buffer in Figure S1a). Under these conditions the electron transfer likely precedes the proton transfer due to the basic media,^[26]^ and a ketyl radical is produced that hydrogenates to the hydroxyl radical. However, due to the large number of HMF molecules and the charge transfer limitation, the second H^+^/e^−^ transfer does not occur. Consequently, the hydroxyl radical quickly reacts with another radical, leading to the pinacol coupling. With a more negative potential, the rate of HMF reduction increases, and therefore the two consecutive H^+^/e^−^ steps could occur, as proposed for furfural.^[22]^ However, the role of H_ads_ on either decreasing the available sites for the HMF adsorption or directly participating in the formation of the BHMF could be not ruled out.

### Electrochemical reduction of HMF modifying the pH and the electrocatalyst

To further investigate the electrochemical reduction of HMF, two different approaches are adopted: (i) slightly acidifying the electrolyte to pH=8.5; and (ii) using a more active HER electrocatalyst at pH=9.2. The electrocatalytic reduction tests are performed under potentiostatic conditions (−0.51 V vs. RHE), since under galvanostatic conditions both the potential applied and the HMF concentration are modified along the test, making the interpretation of the data more complex.

**Figure 8:**
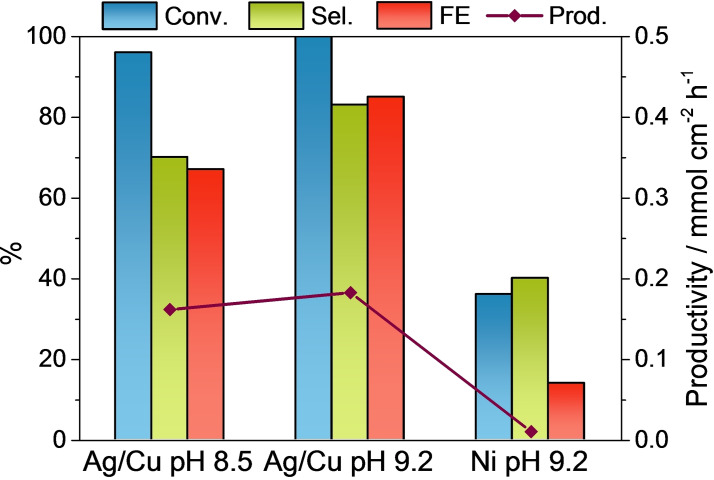
HMF conversion, BHMF selectivity and productivity, and FE obtained in the electrocatalytic reduction at −0.51 V vs. RHE of 0.05 m HMF solutions over AgCu foam at pH 9.2 and 8.5 and over a Ni foam at pH 9.2.

In near‐neutral media (pH=8.5), nearly all the HMF is converted, but the selectivity in BHMF decreases (Figure 8). As expected, the pH modifies the HER. In Figure 1 it is observed that the onset shifts towards less cathodic potentials, and a higher current density is reached at pH=8.5 in the borate electrolyte in comparison to pH=9.2. The HMF reduction also seems to be pH‐dependent, with an onset at around −0.14 V vs. RHE. This behavior could be related to modifications in the proton transfer mechanism that is correlated to the solution pH.^[19]^ Thus, a decrease in the pH to 8.5 may increase the availability of H^+^ for the H^+^/e^−^ transfer but also the hydrogenation through H_ads_.

To promote the activity in the HER without modifying the pH of the electrolyte a Ni electrocatalyst is used. The higher activity of Ni in the HER shifts the onset in the borate electrolyte towards less cathodic potentials (Figure 1). Remarkably, the addition of HMF to the borate buffer does not significantly modify the LSV curve as recently reported for a Ni plate.^[35]^ Despite the absence of the characteristic HMF reduction peak in the voltammogram, the catalyst is active in the reduction of HMF. At −0.51 V vs. RHE it reaches a 36 % conversion, though the selectivity and FE are largely decreased, 40 and 14 %, respectively (Figure 8). The similarities between the LSV in borate and HMF suggest that the reaction pathway taking place over Ni is the electrochemical hydrogenation with H_ads_, through a Langmuir–Hinshelwood mechanism.^[21]^ However, recent computational studies stated that the differences in the reactivity of Ni and Ag are related to the interaction of the molecule with the metals.^[35]^ A stronger interaction for Ni than for Ag favors the hydrogenolysis and the formation of methyl furfuryl alcohol (MFA), which could explain both the decrease in the conversion and selectivity, since the process consumes 4 e^−^ rather than 2 e^−^ required to form BHMF. Unfortunately, in this work we could not quantify the MFA.

## Conclusion

The electrocatalytic reduction of 0.05 m 5‐hydroxymethylfurfural (HMF) solutions (pH=9.2) is feasible at current densities of 40–50 mA cm^−2^ over AgCu foams, achieving an outstanding 0.567 mmol cm^−2^ h^−1^ 2,5‐bishydroxymethylfuran (BHMF) productivity rate. Contrarily, at a low current density, the BHMF selectivity decreases due to the coproduction of the pinacol by electrodimerization. These results are related to the electrode potential required to keep the current density, although it should be also considered that the selectivity is modified with time‐on‐stream due to the consumption of HMF.

To foster the BHMF production with the AgCu catalysts, the potential should be in the hydrogen evolution reaction (HER) window, wherein the HMF reduction is favored over the production of H_2_, although the latter is not avoided. The contribution of the Volmer step, decreasing the available sites for the HMF adsorption, likely improves the selectivity in BHMF. However, the promotion of the HER by modifying either the electrolyte pH or the catalyst, and in turn the formation of H_ads_, provokes a drop in the BHMF selectivity. This behavior is more remarkable by using a Ni foam at pH 9.2 than by decreasing the pH to 8.5 in the experiments with the AgCu foam, likely also due to a change in the interaction of HMF molecule and catalyst. This information together with results obtained from electrochemical impedance spectroscopy, which evidence that HMF reduction is limited by charge transfer, could indicate that the reaction follows a H^+^/e^−^ pathway. However, further work is required to elucidate the reaction mechanism.

The information obtained herein indicates that the current density at which the AgCu electrocatalyst will selectively convert HMF to BHMF is related to the electrode potential required to keep the selected current density value, and in turn to its electroactivity. These outputs could help to develop more selective catalysts, though some more work is required to operate at both higher current densities (>50 mA cm^−2^) and HMF concentrations (>0.05 m).

## Experimental Section

### Materials and chemicals

Cu and Ni foam panels were supplied by Alantum. Chemicals used were sodium hydroxide (≥98 %, Sigma‐Aldrich), boric acid (≥99.5 %, Sigma‐Aldrich), silver nitrate (99.9+%, Alfa Aesar), and 5‐hydroxymethylfurfural (99 %, AVA Biochem). 2,5‐Bis(hydroxymethyl)furan (Toronto Research Chemicals) was used as standard for high‐performance liquid chromatography (HPLC) analysis. All chemicals were used without further purification. Ultrapure water (UPW, 18 MΩ cm) was used for the preparation of all aqueous solutions.

### Preparation of electrocatalysts

Ni and Cu foams were cut from the 1.6 mm thickness and 450 μm cell size foam panels, into pieces of 10 mm ×10 mm (geometric surface area 2.64 cm^2^). Before the use, the electrodes were washed with 2‐propanol, UPW, and 1 m HCl for 5 min to remove surface oxides, and UPW to remove HCl. Ag/Cu electrodes were synthesized by electrodeposition in a single‐compartment three‐electrode cell controlled by a potentiostat/galvanostat Metrohm Autolab PGSTAT204, equipped with NOVA software as reported elsewhere.^[32]^

### Characterization techniques

XRD analysis was carried out directly at the foam specimens using a PANalytical X'Pert diffractometer equipped with a copper anode (*λ*_mean_=0.15418 nm) and a fast X'Celerator detector. Wide‐angle diffractogram was collected over a 2*θ* range from 3 to 80° with a step size of 0.067° and counting time per step of 60.95 s.

The surface morphology of the foam electrodes was examined by SEM/energy‐dispersive X‐ray spectroscopy (EDS) and FE‐SEM/EDS. The SEM/EDS was an EP EVO 50 Series Instrument (EVO ZEISS) equipped with an INCA X‐act Penta FET® Precision EDS microanalysis and INCA Microanalysis Suite Software (Oxford Instruments Analytical). The accelerating voltage was 20 kV, and the spectra were collected in duration 60 s. The FE‐SEM/EDS was a ZEISS Leo 1530 equipped with an INCA EDS microanalysis and INCA Microanalysis Suite Software (Oxford Instruments Analytical). The accelerating voltage was 10 kV, and the EDS spectra were collected during a period of 60 s.

### Electrochemical measurements

Electrochemical measurements were controlled by a potentiostat/galvanostat Metrohm Autolab PGSTAT204, equipped with NOVA software; Cu or Pt wires were attached to the Cu and Ni‐based electrodes, respectively, to enable connection to the potentiostat. A three‐electrode three‐compartment cell, separated by glass frits, was used to perform all the electrochemical measurements. Working electrodes were the electrocatalysts, placed in the central compartment, with the reference electrode (SCE) put in electrolytic contact via a Luggin capillary. Counter electrodes were Pt wires, placed in the side compartments. Electrolytes were 25 mL of 0.5 m borate buffer aqueous solution (pH=9.2) with and without HMF 0.02 and 0.05 m, in the cathode compartment, and 0.5 m borate buffer solution (pH=9.2) with 0.5 m sodium sulfite, in the anode compartment. All potentials were reported vs SCE and RHE [Eq. 1]:

(1) Vvs.RHE=Vvs.SCE+0.244V+0.0591×pH

The cell was thermostated with a water bath at 25 °C. The ohmic drop (*iR*_u_, *i*=current density, *R*_u_=uncompensated resistance) for LSV was corrected after measurements, whereas the constant‐potential electroreductions were measured without compensation. Note that very low *R*_u_ values (≈1–2 Ω) are measured. To avoid the presence of dissolved oxygen, all the solutions were purged with N_2_ before each electrochemical experiment, and a N_2_ flow was kept in the open space of the cell during experiments. In LSV, the potential was scanned from 0 to −1.4 V vs. SCE (from 0.79 to −0.61 V vs. RHE) at a scan rate of 1 mV s^−1^ in the electrolytes without HMF and 5 mV s^−1^ in those with HMF, as reported elsewhere.^[32]^ Electrocatalytic reductions were performed: (i) galvanostatically at current densities of 5, 10, 25, 40, and 50 mA cm^−2^ recording the electrode potential (note that the area used to calculate the surface area corresponds to both faces of the foam piece); ii) potentiostatically in the −1.1 to −1.5 V vs. SCE range (−0.31 to −0.71 V vs. RHE). The solution was kept under stirring, with a magnetic bar, at 1000 rpm. The catalytic cycle started with a sequence of LSV in borate and borate plus HMF, followed by electrolysis at constant current or potential and then the first two LSVs were repeated. Several cycles could be performed over the same electrocatalyst, either modifying or keeping constant the current or potential applied. The reactions were carried out under total HMF conversion conditions, which were obtained through the transfer of the charge necessary to convert all HMF in solution into BHMF (i. e., through a 2 e^−^ process) assuming 100 % FE. Some selected reactions were also performed at a short time. At the end, the solutions were collected and analyzed with HPLC. All the measurements were performed in triplicates. The geometric surface areas of the electrodes were considered to determine the current densities.

EIS was measured in the same three‐electrode three compartments cell with a Biologic VSP‐300 multichannel bi‐potentiostat. EIS experiments were performed in borate solution with and without HMF 0.02 and 0.05 m from 100 kHz to 100 mHz with amplitude of 20 mV, between −1.10 (−0.31 V vs. RHE) and −1.30 V vs. SCE (−0.51 V vs. RHE) potential.

### Product analysis

An HPLC Agilent 1260 Infinity Series equipped with a Cortecs T3 2.4 μm (4.6×100 mm) was used to analyze and quantify the reaction products. The instrument operates at 30 °C, with an autosampler (injection volume 1 μL) and a diode‐array detector set at 284 nm for the identification of HMF and 223 nm for the identification of BHMF. The analyses were performed with gradient elution in three steps: isocratic conditions for 6 min, with eluent composed of CH_3_CN/H_2_O (10 : 90 *v*/*v*); gradient elution for 5 min until a CH_3_CN/H_2_O (50 : 50) elution ratio was obtained; gradient elution for 4 min until a CH_3_CN/H_2_O (70 : 30) elution ratio was obtained. The flow rate was 0.7 mL min^−1^.

Conversion (*X*_HMF_), selectivity (*S*_BHMF_), FE, BHMF productivity, and carbon balance were calculated with the following Equations (2–6:

(2) $$X_{HMF\;}\left[\%\right]=\frac{{mol}_{HMF\;consumed}}{{mol}_{HMF\;initial\;}}\times 100$$

(3) $$S_{BHMF}\;\left[\%\right]=\frac{{mol}_{BHMF\;formed}}{{mol}_{HMF\;consumed}}\times 100$$

(4) $$FE\;\left[\%\right]=\frac{{mol}_{BHMF\;formed}}{total\;charge\;passed/\left(2F\right)}\times 100$$

(5) $$BHMF\;productivity=\;\frac{m{mol}_{BHMF\;formed}}{reaction\;time\;\left(h\right)\;{}^{*}area\;\left(cm^{2}\right)}$$

where F is the Faraday constant. The area corresponds to the geometric area of electrodes (i. e. 2.64 cm^2^).

(6) $$C-\mathrm{balance}=\;\frac{m{mol}_{BHMF\;formed}+\;m{mol}_{HMF\;residual}}{m{mol}_{HMF\;initial}}$$

All the measurements at constant current density were repeated three times; the standard deviation of the values reported in this work was lower than 1 %.

In the HPLC chromatograms a peak close to the one for BHMF was detected, which was related to 5,5’‐bis(hydroxymethyl)hydrofuroin, in agreement with gas chromatography mass spectrometry (GC‐MS) and electrospray ionization mass spectrometry (ESI‐MS) analyses previously reported.^[16]^ The area of the peak was used to estimate its formation during the electroreduction experiments over the different investigated electrocatalysts since we were not able to find a standard to quantify it.

## Conflict of interest

The authors declare no conflict of interest.

1

## Supporting information

As a service to our authors and readers, this journal provides supporting information supplied by the authors. Such materials are peer reviewed and may be re‐organized for online delivery, but are not copy‐edited or typeset. Technical support issues arising from supporting information (other than missing files) should be addressed to the authors.

Supporting Information

## Data Availability

Research data are not shared.

## References

[1] A. A. Rosatella , S. P. Simeonov , R. F. M. Frade , C. A. M. Afonso , Green Chem. 2011, 13, 754–793.

[2] C. Xu , E. Paone , D. Rodríguez-Padrón , R. Luque , F. Mauriello , Chem. Soc. Rev. 2020, 49, 4273–4306. 32453311 10.1039/d0cs00041h

[3] S. Chen , R. Wojcieszak , F. Dumeignil , E. Marceau , S. Royer , Chem. Rev. 2018, 118, 11023–11117. 30362725 10.1021/acs.chemrev.8b00134

[4] T. S. Hansen , K. Barta , P. T. Anastas , P. C. Ford , A. Riisager , Green Chem. 2012, 14, 2457–2461.

[5] T. Pasini , A. Lolli , S. Albonetti , F. Cavani , M. Mella , J. Catal. 2014, 317, 206–219.

[6] S. A. Akhade , N. Singh , O. Y. Gutiérrez , J. Lopez-Ruiz , H. Wang , J. D. Holladay , Y. Liu , A. Karkamkar , R. S. Weber , A. B. Padmaperuma , M.-S. Lee , G. A. Whyatt , M. Elliott , J. E. Holladay , J. L. Male , J. A. Lercher , R. Rousseau , V.-A. Glezakou , Chem. Rev. 2020, 120, 11370–11419. 32941005 10.1021/acs.chemrev.0c00158

[7] Y. Kwon , K. J. P. Schouten , J. C. van der Waal , Ed de Jong , Marc T. M. Koper , ACS Catal. 2016, 6, 6704–6717.

[8] O. Simoska , Z. Rhodes , S. Weliwatte , J. R. Cabrera-Pardo , E. M. Gaffney , K. Lim , S. D. Minteer , ChemSusChem 2021, 14, 1674–1686. 33577707 10.1002/cssc.202100139

[9] P. Nilges , U. Schröder , Energy Environ. Sci. 2013, 6, 2925–2931.

[10] Y. Kwon , Y. Y. Birdja , S. Raoufmoghaddam , M. T. M. Koper , ChemSusChem 2015, 8, 1745–1751. 25908308 10.1002/cssc.201500176

[11] Y.-R. Zhang , B.-X. Wang , L. Qin , Q. Li , Y.-M. Fan , Green Chem. 2019, 21, 1108–1113.

[12] J. J. Roylance , K.-S. Choi , Green Chem. 2016, 18, 2956–2960.

[13] J. J. Roylance , T. W. Kim , K.-S. Choi , ACS Catal. 2016, 6, 1840–1847.

[14] L. Zhang , F. Zhang , F. C. Michel Jr. , A. C. Co , ChemElectroChem 2019, 6, 4739–4749.

[15] X. H. Chadderdon , D. J. Chadderdon , T. Pfennig , B. H. Shanks , W. Li , Green Chem. 2019, 21, 6210–6219.

[16] G. Sanghez de Luna , P. H. Ho , A. Lolli , F. Ospitali , S. Albonetti , G. Fornasari , P. Benito , ChemElectroChem 2020, 7, 1238–1247.

[17] R. Kloth , D. V. Vasilyev , K. J. J. Mayrhofer , I. Katsounaros , ChemSusChem 2021, 14, 5245–5253. 34549892 10.1002/cssc.202101575 PMC9298403

[18] U. Sanyal , S. F. Yuk , K. Koh , M.-S. Lee , K. Stoerzinger , D. Zhang , L. C. Meyer , J. A. Lopez-Ruiz , A. Karkamkar , J. D. Holladay , D. M. Camaioni , M.-T. Nguyen , V.-A. Glezakou , R. Rousseau , O. Y. Gutiérrez , J. A. Lercher , Angew. Chem. Int. Ed. 2021, 60, 290–296; 10.1002/anie.202008178 PMC7821193 32770641

[19] D. C. Cantu , A. B. Padmaperuma , M.-T. Nguyen , S. A. Akhade , Y. Yoon , Y.-G. Wang , M.-S. Lee , V.-A. Glezakou , R. Rousseau , M. A. Lilga , ACS Catal. 2018, 8, 7645–7658.

[20] X. H. Chadderdon , D. J. Chadderdon , J. E. Matthiesen , Y. Qiu , J. M. Carraher , J.-P. Tessonnier , W. Li , J. Am. Chem. Soc. 2017, 139, 14120–14128. 28903554 10.1021/jacs.7b06331

[21] C. J. Bondue , M. T. M. Koper , J. Catal. 2019, 369, 302–311.

[22] J. Anibal , B. Xu , ACS Catal. 2020, 10, 11643–11653;

[23] X. Shang , Y. Yang , Y. Sun , Green Chem. 2020, 22, 5395–5401.

[24] J. Ludvík in *Organic Electrochemistry Revised and Expanded* (Eds. O. Hammerich, B. Speiser) CRC Press, Boca Raton, US, **2016**, Ch. 31.

[25] L. A. Diaz , T. E. Lister , C. Rae , N. D. Wood , ACS Sustainable Chem. Eng. 2018, 6, 8458–8467.

[26] P. Zuman , Electroanalysis 2006, 18, 131–140.

[27] Y. Kwon , E. de Jong , S. Raoufmoghaddam , M. T. M. Koper , ChemSusChem 2013, 6, 1659–1667. 23857762 10.1002/cssc.201300443

[28] H. Liu , T.-H. Lee , Y. Chen , E. W. Cochran , We. Li , ChemElectroChem 2021, 8, 2817–2824.

[29] Z. Li , S. Kelkar , C. H. Lam , K. Luczek , J. E. Jackson , D. J. Miller , C. M. Saffron , Electrochim. Acta 2012, 64, 87–93.

[30] C. Ampelli , C. Genovese , D. Cosio , S. Perathoner , G. Centi , Chem. Eng. Trans. 2019, 74, 1285–1290.

[31] S. Jung , E. J. Biddinger , Energy Technol. 2018, 6, 1370–1379.

[32] G. Sanghez de Luna , P. H. Ho , A. Sacco , S. Hernández , J.-J. Velasco-Vélez , F. Ospitali , A. Paglianti , S. Albonetti , G. Fornasari , P. Benito , ACS Appl. Mater. Interfaces 2021, 13, 23675–23688. 33974392 10.1021/acsami.1c02896 PMC8289175

[33] L. Delmondo , J. A. Muñoz-Tabares , A. Sacco , N. Garino , G. Massaglia , M. Castellino , G. P. Salvador , C. F. Pirri , M. Quaglio , A. Chiodoni , Phys. Chem. Chem. Phys. 2017, 19, 28781–28787. 29048084 10.1039/c7cp05091g

[34] M. A. Farkhondehfal , S. Hernández , M. Rattalino , M. Makkee , A. Lamberti , A. Chiodoni , K. Bejtka , A. Sacco , F. C. Pirri , N. Russo , Int. J. Hydrogen Energy. 2020, 45, 26458–26471.

[35] D. K. Lee , S. R. Kubota , A. N. Janes , M. T. Bender , J. Woo , J. R. Schmidt , K.-S. Choi , ChemSusChem 2021, 14, 4563–4572. 34378355 10.1002/cssc.202101037

