# Supplementary material for: Insights into the Electrochemical Reduction of 5‐Hydroxymethylfurfural at High Current Densities
Source: ChemSusChem. 2022 Mar 3;15(13):e202102504. doi: 10.1002/cssc.202102504 (PMC9400883; doi:10.1002/cssc.202102504)
Supplement: Supplementary file 1 — Supporting Information [file CSSC-15-0-s001.pdf]

ChemSusChem

Supporting Information

Insights into the Electrochemical Reduction of 5-Hydroxymethylfurfural at High Current Densities

Giancosimo Sanghez de Luna, Adriano Sacco, Simelys Hernandez, Francesca Ospitali, Stefania Albonetti, Giuseppe Fornasari, and Patricia Benito*This publication is part of a collection of invited contributions focusing on “Green Conversion of HMF”. Please visit to view all contributions.© 2022 The Authors. ChemSusChem published by Wiley-VCH GmbH. This is an open access article under the terms of the Creative Commons Attribution License, which permits use, distribution and reproduction in any medium, provided the original work is properly cited.

Table S1. Carbon balance of the electrochemical reduction of 0.05 M HMF (pH = 9.2) at different current densities over AgCu foam.

Current density / mA cm^{-2}	Carbon Balance
**5**	0.712
**10**	0.699
**15**	0.797
**25**	0.810
**40**	0.874
**50**	0.882

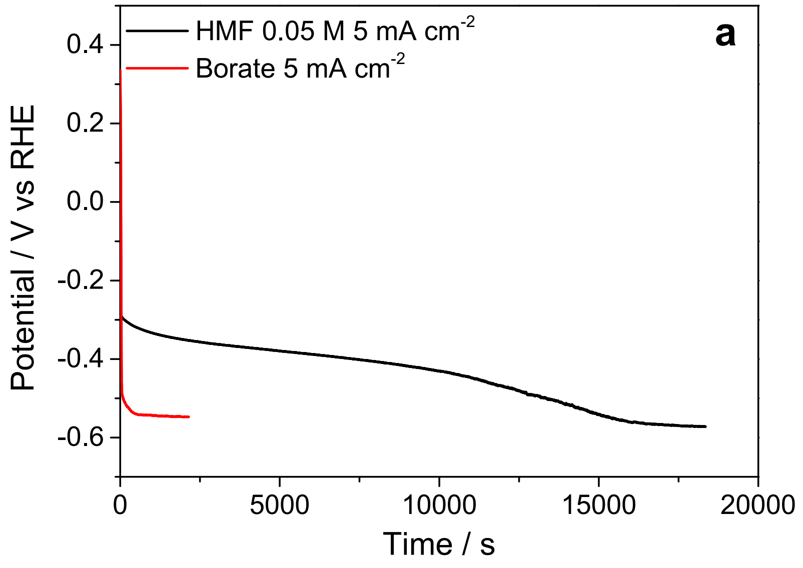

Figure S1a. Evolution of the potential applied with the reaction time during a test at 5 mA cm^{-2} in an HMF containing and HMF free electrolyte (only borate). In absence of HMF the potential suddenly moves to -0.51 V vs RHE, which is the value reached at the end of the test in the HMF containing electrolyte.

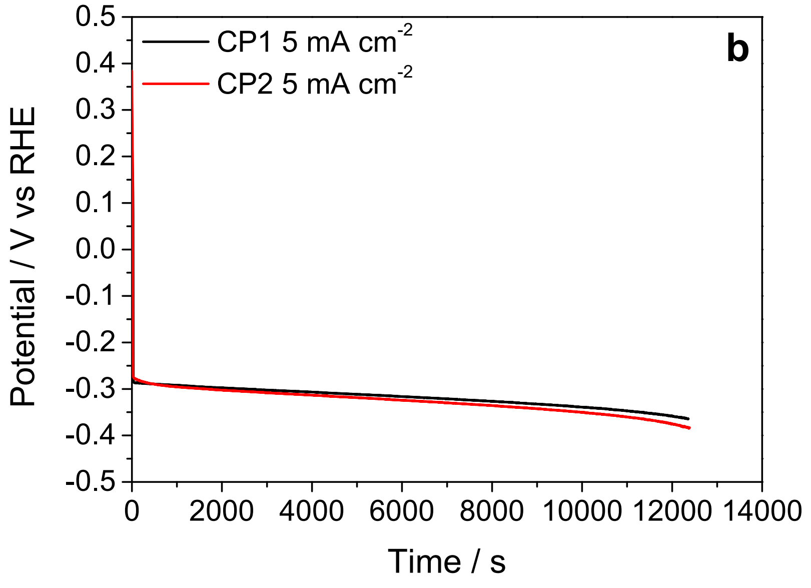

	Conv. HMF / %	Sel. BHMF / %	FE / %	Hydrof/Conv	Productivity / mmol cm^{-2} h^{-1}
**CP1 5 mA cm^{-2} stopped**	88	56	76	1125	0.0709
**CP2 5 mA cm^{-2} stopped**	88	57	76	1170	0.0708

Figure S1b. Evolution of the potential applied with the reaction time during a test at 5 mA cm^{-2} (AgCu foam, 0.05 M HMF, pH = 9.2) stopped before the contribution of the concentration polarization becomes significant. The catalytic results in the table show that the HMF conversion and BHMF selectivity values are lower than for the test accumulating the full charge.

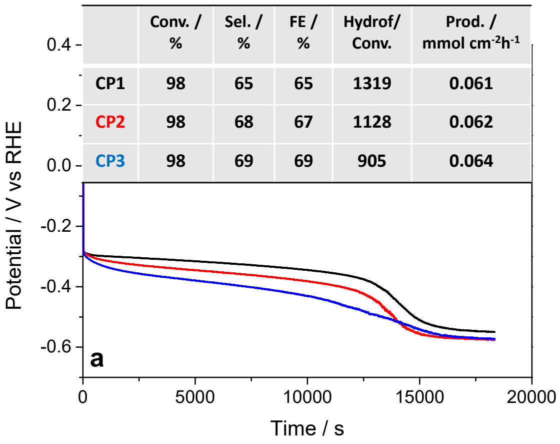

	Conv. / %	Sel. / %	FE / %	Hydrof/ Conv.	Prod. / mmol cm^{-2}h^{-1}
**CP1**	**98**	**65**	**65**	**1319**	**0.061**
**CP2**	**98**	**68**	**67**	**1128**	**0.062**
**CP3**	**98**	**69**	**69**	**905**	**0.064**

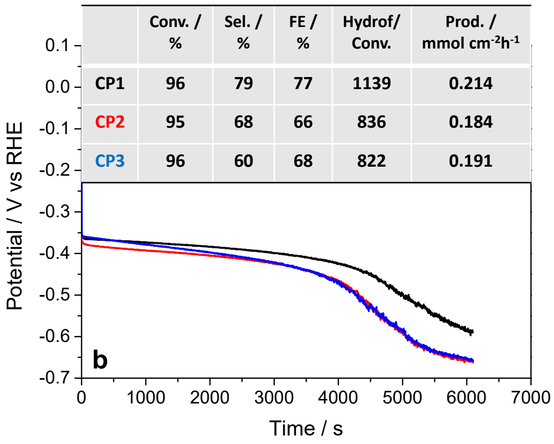

	Conv. / %	Sel. / %	FE / %	Hydrof/ Conv.	Prod. / mmol cm^{-2}h^{-1}
**CP1**	**96**	**79**	**77**	**1139**	**0.214**
**CP2**	**95**	**68**	**66**	**836**	**0.184**
**CP3**	**96**	**60**	**68**	**822**	**0.191**

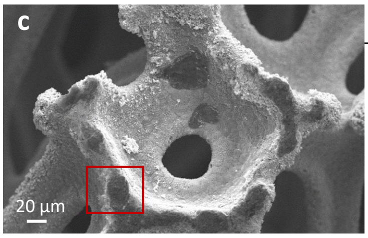

Element	Atomic / %
C	49.65
O	10.37
Cu	36.09
Ag	3.89

Figure S2. Catalytic activity and evolution of the potential in three consecutive tests at 5 mA cm^{-2} (a) and 15 mA cm^{-2} (b). SEM image of an Ag/Cu foam after the tests at 5 mA cm^{-2} (c).

Table S2. Electrocatalytic performance of a AgCu foam in the reduction of a 0.02 M HMF solution at 20 and 40 mA cm^{-2}

	Conv. HMF / %	Sel. BHMF / %	FE / %	Productivity / mmol cm^{-2} h^{-1}
**20 mA cm^{-2}**	81	87	74	0.278
**40 mA cm^{-2}**	61	92	59	0.439

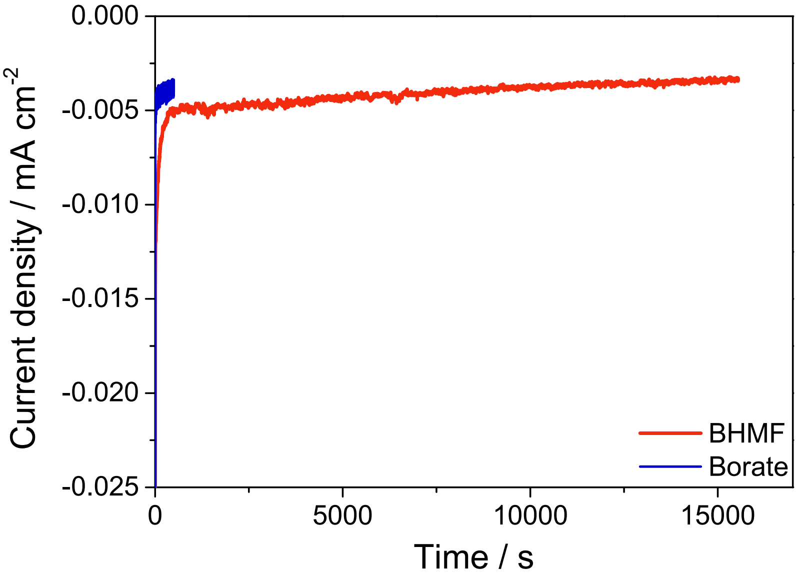

Figure S3. Evolution of the current density during the test with a 0.05 M BHMF solution pH = 9.2 at -0.51 V vs RHE

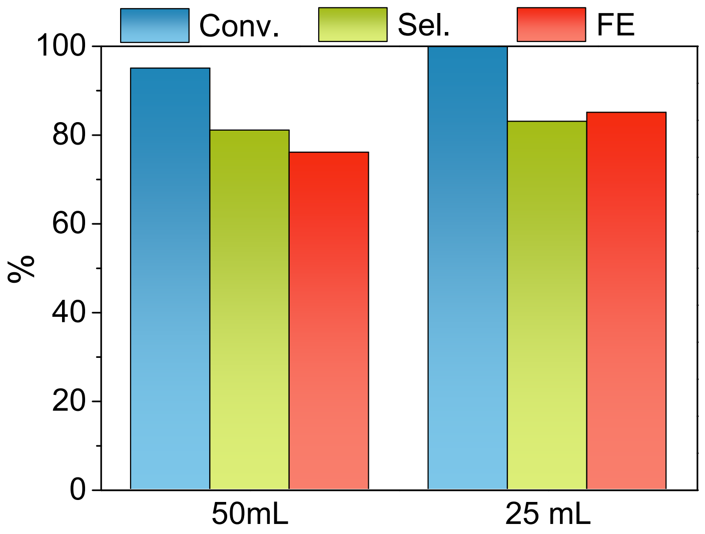

Figure S4. Conversion of HMF, Selectivity in BHMF and FE obtained during the electroreduction of 50 and 25 mL of a 0.05 M HMF solution for an AgCu foam at -0.51 V vs RHE.
